# RNA-Binding Protein Trim71 Controls Epicardial Cell Migration

**DOI:** 10.3390/jcdd13060237

**Published:** 2026-05-31

**Authors:** Juan Manuel Castillo-Casas, Carlos García-Padilla, Rita Carmona, Estefanía Lozano-Velasco, Diego Franco

**Affiliations:** 1 Cardiovascular Development Group, Department of Experimental Biology, Faculty of Experimental Sciences, University of Jaen, 23071 Jaen, Spain; jmcasas@ujaen.es (J.M.C.-C.); cgpadill@ujaen.es (C.G.-P.); evelasco@ujaen.es (E.L.-V.); 2 Department of Human Anatomy, Legal Medicine and History of Science, Faculty of Medicine, University of Málaga, 29071 Málaga, Spain; rita@uma.es

**Keywords:** Trim71, epicardium, cell migration, microRNAs, post-transcriptional regulation

## Abstract

The epicardium is an embryonic tissue layer essential for heart morphogenesis, providing progenitor cells and regulatory signals that support myocardial growth and coronary vessel formation. Epicardial cells arise from the proepicardium (PE) and spread over the myocardium to form the embryonic epicardium (EE), a transition that requires tight coordination between proliferation, migration, and lineage priming. However, the molecular mechanisms controlling this developmental timing remain incompletely understood. Here, we identify *Trim71* as a key regulator of epicardial cell behaviour during the PE-to-EE transition. *Trim71* is enriched in the PE and subsequently downregulated as cells acquire migratory competence. Functional analyses show that loss of *Trim71* function decreases proliferation while promoting migration, as well as inducing the expression of epicardial commitment markers, suggesting that *Trim71* is a controller of a progenitor-like state. We further demonstrate that *Trim71* is necessary for these processes through a reciprocal feedback loop with the microRNAs *let-7c* and *miR-30c*. Our findings establish *Trim71* as a temporal gatekeeper that coordinates the balance between progenitor maintenance and migration during early epicardial development. This *Trim71*-miRNAs axis constitutes a novel post-transcriptional layer of regulation that ensures the correct timing of epicardium development during cardiogenesis.

## 1. Introduction

The embryonic epicardium (EE) originates from an extracardiac primordium known as the proepicardium (PE) [1]. In the mouse, the PE emerges at embryonic day (E) 9.5 as a cluster of mesothelial cells located between the sinus venosus and the hepatic primordium, being a highly conserved structure among vertebrates [1,2,3,4,5,6,7]. Following its formation, PE cells migrate to the surface of the naked heart, which is only composed of myocardium and endocardium at this stage, through cellular bridges or via cellular vesicles, progressively covering the heart and giving rise to the third cardiac layer, the EE, by E10.5 [1]. Once established, a subset of epicardial cells undergoes an epithelial-to-mesenchymal transition (EMT) and invades the myocardium, giving rise to epicardial-derived cells (EPDCs). In mice, this process occurs around E11.5 and is characterized by the loss of epithelial features and the acquisition of migratory and invasive properties similar to those of multipotent mesenchymal cells [8,9]. EPDCs constitute a major progenitor source for several cardiac cell types, including cardiac fibroblasts (CFs), smooth muscle cells (SMCs), and pericytes (PCs) [10,11]. Beyond its role as a progenitor cell source, the EE functions as a crucial signaling hub capable of inducing cardiomyocyte (CM) cell cycle re-entry, promoting angio-vasculogenesis, recruiting macrophages, and contributing to extracellular matrix deposition. These functions are essential for proper myocardial support and morphogenetic processes such as trabeculation and myocardial compaction [12,13,14]. Moreover, the epicardium can be reactivated after cardiac injury [12]. Consistent with its central role, defects in epicardial development result in a wide range of cardiac abnormalities, including valve malformations, myocardial non-compaction, fibrosis-associated arrhythmias, and cardiomyopathies, highlighting the functional importance of the epicardium [15,16,17,18].

Mammalian heart development is a highly coordinated process requiring precise regulation of cell differentiation, migration, and proliferation across multiple cardiac lineages. These events are tightly controlled by transcriptional programs governed by several transcription factors (TFs) [19]. In the epicardium, TFs such as *Wt1* and *Tbx18* have been identified as key regulators of essential processes, including EMT and EPDC differentiation [20,21,22,23]. However, transcriptional regulation alone cannot fully account for the dynamic changes in gene expression that accompany epicardial development. Beneath this layer of control lies post-transcriptional regulation, which is mediated in part by microRNAs (miRNAs). While a transcriptional network provides the framework for epicardial identity, post-transcriptional mechanisms act as essential fine-tuners that ensure the precise timing of these cellular transitions. miRNAs are small non-coding RNAs of approximately 22 nucleotides that bind to the 3′ untranslated regions (3′UTRs) of target mRNAs, leading to translational repression or mRNA degradation [24]. miRNA biogenesis begins in the nucleus and is completed in the cytoplasm, where mature miRNAs associate with the RNA-induced silencing complex (RISC) and Argonaute (Ago) proteins to guide them to their target transcripts [25,26,27,28]. Despite their known roles in multiple developmental contexts, post-transcriptional regulatory mechanisms in epicardial development remain poorly explored. Notably, epicardial deletion of Dicer, an enzyme essential for miRNA maturation, results in postnatal lethality, underscoring the functional importance of miRNAs in epicardial biology [29].

Although considerable progress has been made in characterizing the cellular transition from the PE to the EE, the molecular mechanisms governing this process remain poorly understood. To gain further insights, we previously performed RNA-seq analyses of the PE and EE, identifying distinct sets of differentially expressed mRNAs and miRNAs between these two developmental stages [30]. Among the transcripts enriched in the PE, *Trim71* emerged as a prominent candidate. *Trim71* is an RNA-binding protein and translational repressor known to promote cell proliferation, inhibit differentiation, and regulate cell cycle progression during early embryonic and neural development [31]. In addition, *Trim71* has been shown to modulate miRNA biogenesis, further supporting its role as a post-transcriptional regulator [32]. However, its function in epicardial development has not yet been investigated. Of note, among the miRNAs upregulated in the EE, *let-7c* and *miR-30c* were identified as potential regulators of *Trim71*, since there are binding sites for both of them within the *Trim71* 3′UTR. These findings suggest the existence of an miRNA-dependent regulatory axis controlling *Trim71* expression during the PE-to-EE transition. In this study, we investigate the functional relevance of the *let-7c/miR-30c*–*Trim71* regulatory axis during epicardial development. Our data demonstrate that coordinated regulation of *Trim71* by *let-7c* and *miR-30c* modulates embryonic epicardial cell migration, linking microRNA-mediated post-transcriptional control to a key cellular process required for epicardial maturation. Together, these findings identify *Trim71* as a previously unrecognized regulator of epicardial biology and highlight the importance of miRNA-driven regulatory mechanisms in heart development.

## 2. Materials and Methods

### 2.1. Mouse Lines and Tissue Collection

All experimental procedures were conducted in accordance with the guidelines and approved consent of the Ethics Committee of the University of Jaén and Andalusian Regional Government (14/03/2022/038). Time-pregnant CD1 female mice were euthanized to collect embryos at developmental stages E9.5 to E10.5. For E9.5 samples, the proepicardium was manually dissected, pooled, and stored in a lysis buffer at −80 °C for subsequent RNA isolation. Embryonic epicardial cells were harvested from ventricular explants as previously described by Castillo-Casas et al. [30]. Additionally, the adult epicardium (180 days; n = 6 experimental replicates) was manually dissected from adult CD1 female mice and stored in buffer lysis at −80 °C until further processing.

### 2.2. Cell Lines

Two cell lines, the mouse embryonic epicardial cell line MEC1 (Sigma-Aldrich, St. Louis, MO, USA, SCC187) and 3T3 cells (Merck, Darmstadt, Germany, 93061524), were used in this study. Each cell line was cultured following the manufacturer’s recommendations for 24 h at 37 °C in a humidified atmosphere of 5% CO_2_.

### 2.3. microRNA, siRNA, and Plasmid Transfections

For experimental procedures, cells were seeded 24 h before transfection at a density of 4 × 10^4^ cells per well in 24-well plates or 10^4^ cells in glass-bottom dishes for confocal analysis as previously described with lipofectamine 2000 (Thermo Fisher, Waltham, MA, USA, 11668019) [33]. For miRNA overexpression, cells were transfected with 50 nM of pre-miRNAs (Ambion, Austin, TX, USA). For *Trim71* silencing, a concentration range of 60–80 nM of siRNA was initially tested, with the most efficient dose selected for subsequent experiments. Detailed siRNA sequences are provided in Supplementary Table S1. A scramble siRNA was used as a negative control to confirm that siRNA transfection itself did not affect gene expression (Supplementary Figure S1). For the luciferase assay, 200 ng/mL of the pmiR-Report-pGLuc plasmid was co-transfected with 50 nM of the corresponding pre-miRNA let7-c and miR-30c, respectively. Transfection efficiencies were validated by RT-qPCR in preliminary experiments, adjusting the doses for each condition (Supplementary Figure S2A). Then, 24 h post-transfection, cells were processed either for RT-qPCR or immunofluorescence (IFC) analyses. Negative controls treated with Lipofectamine 2000 were run in parallel. To perform IFC analyses, the cells were fixed in 4% PFA for 15 min at room temperature, rinsed two times in PBS for 5 min, and stored in PBS at 4 °C. For RT-qPCR analysis, cells and explant epicardial outgrowths were collected and stored at −80 °C until further processing.

### 2.4. RNA Isolation and qPCR

All RT-qPCR experiments were performed in accordance with the MIQE guidelines [34]. Briefly, total RNA isolation was performed with the ReliaPrep™ RNA Miniprep Systems kit (Promega, Madison, WI, USA) according to the manufacturer’s instructions. For mRNA expression measurements, 500 ng of total RNA was used for retro-transcription with PrimeScript™ RT Master Mix (Takara, Kyoto, Japan), and the resulting cDNA was diluted 1:5. For microRNA expression analyses, 20 ng of total RNA was used for retro-transcription with miRCURY LNA RT Kit (Qiagen, Venlo, The Netherlands) and the resulting cDNA was diluted 1:40. Non-reverse transcribed controls (no-RT) were performed for each sample to exclude genomic contamination, yielding no signal amplification in all cases. Quantitative Real-time PCR experiments were performed using 2 μL of cDNA, GoTaq qPCR Master Mix (Promega, Madison, WI, USA) with specific primer sets as described in Supplementary Table S1. All qPCRs were performed using a CFX384TM thermocycler (Bio-Rad, Hercules, CA, USA) following the manufacturer’s recommendations. The mRNA thermal cycling conditions consisted of 95 °C for 30 s (initial denaturation), followed by 40 cycles of 95 °C for 5 s (denaturation); 60 °C for 10 s (annealing); and 75 °C for 7 s (extension). For microRNAs, the protocol included a cycle of 95 °C for 10 min (initial denaturation), followed by 40 cycles of 95 °C for 5 s (denaturation) and 60 °C for 1 min (annealing and extension). Melting curve analysis for both cases was determined by an initial step of 95 °C for 5 s, followed by 0.5 °C increments for 7 s from 65 °C to 95 °C. The relative expression levels of each gene were calculated using Livak & Schmittgen [35] with *Gapdh* as the internal control for mRNA expression analyses and 5S for microRNA expression analyses. Each qPCR reaction was performed in technical triplicate and repeated for at least three distinct biological samples to obtain representative means.

### 2.5. Cell Migration Assay

Cell migration was analyzed using a wound-healing (scratch) assay, as previously described by Ascione et al. [36]. MEC1 epicardial cells were plated on 24-well plates at a density of 5 × 10^4^ cells per well and cultured until reaching 90–100% confluence. After 24 h of transfection, cell monolayers were manually scratched using a sterile p200 pipette tip to create a linear wound. After scratching, cells were washed twice with PBS to remove cellular debris and replaced with a fresh medium. Wound healing was monitored by capturing images at 0 h, 6 h, 12 h, and 24 h using an inverted microscope (10 wells per condition; n = 3 experimental replicates). The scratches were quantified using Image J software (version V2.9.0), and the migration rate was expressed as the percentage of wound closure relative to the initial area at 0 h.

### 2.6. Luciferase Assay

The murine *Trim71* 3′UTR sequence was retrieved from the Ensembl Genome Browser database, and mature miRNA sequences (*let-7c-5p* and *miR-30c-5p*) were obtained from miRBase. Sequence analysis and visualization were performed in R using the packages ggplot2, tibble, and dplyr, and putative miRNA binding sites were identified based on canonical seed-sequence complementarity and corroborated with TargetScan predictions. The target fragment was amplified using the iPROOF High-Fidelity PCR kit (Bio-Rad 1725330) and cloned into the pmiR-Report-pGLuc report vector. For the reporter assays, 3T3 cells were co-transfected with the pmiR-Trim71-3′UTR-pGLuc construct, the corresponding miRNA precursors, *let-7c* and *miR-30c* mimics, and pCLuc control vector for internal normalization in 3T3 cells. Luciferase activity was measured 24 h after transfection. *Gaussia* luciferase activity was quantified by the Pierce Gaussia Luciferase Flash Assay Kit (Thermo Fisher Scientific, Waltham, MA, USA, 16159), while normalization was performed by measuring *Cypridina* luciferase activity with the Pierce Cypridina Luciferase Flash Assay Kit (Thermo Fisher Scientific 16169). In all cases, transfections were carried out in triplicate (n = 3 experimental replicates). Atf6 and Raldh2 3′UTRs were used in parallel as positive and negative controls, respectively, for the luciferase assay (Supplementary Figure S2B) [37,38].

### 2.7. Immunofluorescence and Confocal Scanning Laser Microscopy Analyses

Immunofluorescence analyses were performed as previously reported by Bonet et al. [39]. Briefly, control and experimental cell cultures were collected after the corresponding treatment, rinsed in PBS for 5 min at room temperature, and fixed with 4% PFA at room temperature for 15 min. After fixation, the samples were rinsed three times in PBS (10 min each) at room temperature and then permeabilized with 0.02% Triton X-100, 50 nM NH4Cl, and PBS solution for 10 min at room temperature. Non-specific binding sites were blocked with 0.2% gelatin solution (Sigma-Aldrich), which was applied twice for 10 min. As the primary antibody, anti-phospho-histone H3 (pHH3, Abcam, Cambridge, United Kingdom, AB5176), anti-Wt1 (Abcam, ab89901), and anti-Nkx2.5 (Santa Cruz Biotechnology, Dallas, TX, USA, N19) in blocking solution were applied overnight at 4 °C. Cell cultures were rinsed 3 times in PBS for 10 min and incubated with secondary antibody donkey anti-rabbit Alexa Fluor 594 (Thermo A2107) at a 1:200 dilution for 30 min at room temperature. Nuclei were counterstained with DAPI at 1:1000 dilution (Sigma #10236276001) for 15 min at room temperature and rinsed twice in PBS for 5 min each. Each immunofluorescence assay was performed in technical triplicate and repeated at least six times on distinct biological samples to obtain representative means. The percentage of pHH3^+^ cells was calculated in relation to total DAPI^+^ cells in the corresponding region of interest (ROI). For cell size assay and morphology analysis, F-actin filaments were stained using Phalloidin-iFluor 488 Reagent (Abcam ab176753) according to the manufacturer’s instructions. A total of 80 cells per condition were measured with imageJ software using the freehand tool to mark the shape of the cell and adjusting the measurement with the scale bar. Fluorescent histograms for Nkx2.5 and Wt1 signal were measured using the raw integrated density of each antibody and normalized per DAPI raw integrated density of each picture (i.e., ROI). Cells were stored in PBS in the dark at 4 °C until imaging. Confocal images were acquired using a Leica TCS SP5 II confocal scanning laser microscope (Leica Microsystems, Wetzlar, Germany).

### 2.8. Statistical Analysis

Statistical analyses of datasets were performed using GraphPad Prism software 11.0.0 (GraphPad Software, Inc., San Diego, CA, USA). For comparison between two groups, an unpaired Student’s *t*-test was used. Significance levels or *p*-values are stated in each corresponding figure legend. *p*-value < 0.05 was considered statistically significant.

## 3. Results

### 3.1. Trim71 Sustains Cell Proliferation While Restraining Migration in Epicardial Cells

**Figure 1:**
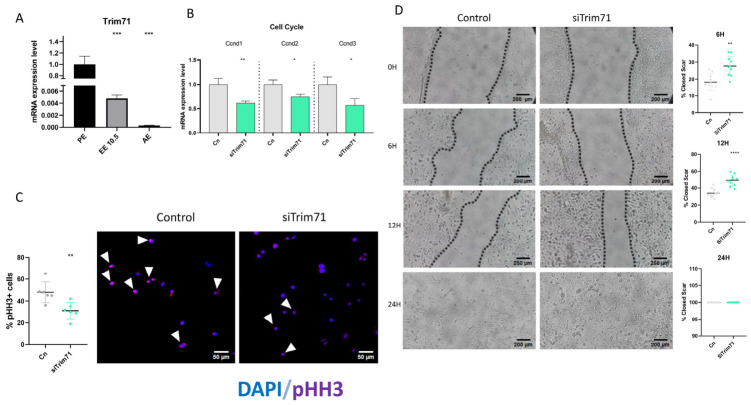
(**A**) RT–qPCR analysis of *Trim71* expression in proepicardium (PE), embryonic epicardium (EE), and adult epicardium (AE) (n = 3), showing significantly higher expression in PE compared with later stages. (**B**) RT–qPCR analysis of cell cycle gene markers (*Ccnd1*, *Ccnd2*, and *Ccnd3*) in Mec1 cells transfected with control siRNA (Cn) or siRNA targeting *Trim71* (si*Trim71*) (n = 3), indicating reduced expression upon *Trim71* knockdown. (**C**) Quantification of the percentage of phospho-histone H3 (pHH3)-positive cells in control and si*Trim71*-transfected Mec1 cells (n = 6), revealing decreased proliferative activity after *Trim71* inhibition. Scale bars represent 50 microns. (**D**) Scratch wound-healing assay in Mec1 cells transfected with control or si*Trim71*. Percentage of wound closure was quantified at 6-, 12-, and 24-h post-scratch (n = 10 fields per condition), showing increased migratory capacity following *Trim71* silencing. Data are presented as mean ± SEM. Scale bars represent 200 microns. Statistical significance is indicated (* *p* < 0.05, ** *p* < 0.01, and *** *p* < 0.001, **** *p* < 0.0001) (Cn: control).

The TRIM-NHL family of E3 ubiquitin ligases is evolutionarily conserved in mammals, and it has been described as a regulator of development and differentiation, acting primarily as a translational repressor [31]. For the first time in the context of epicardial development, our research group identified *Trim71* as a differentially expressed gene between the PE and EE at E10.5 through RNA-seq analysis [30]. RT-qPCR analysis confirmed that *Trim71* is highly expressed in the PE, while it is decreased in embryonic epicardial cells at E10.5 (EE 10.5) and becomes undetectable in adult epicardial cells (AE) (Figure 1A). Importantly, embryonic and adult epicardial cells displayed an enrichment of epicardial markers *Upk3b*, *Upk1b*, *Msln*, and *Pdpn*, as expected (Supplementary Figure S3A), although it is important to note that residual contamination for the underlying myocardium cannot be fully discarded.

*Trim71* has been previously described as a cell proliferation triggering factor [40]. Consistent with this role, we observed that *Trim71* loss of function in the Mec1 epicardial cell line resulted in the reduced expression of *Ccnd1*, *Ccnd2*, and *Ccnd3* cell cycle markers (Figure 1B). These RT-qPCR analyses were further supported by IFC of pHH3 showing a significant decrease in the percentage of proliferative cells after *Trim71* inhibition (Figure 1C). These findings indicate that *Trim71* is required to maintain the proliferative state of epicardial cells. Since cell migration is also a key process during the transition from the PE to the EE, we investigated whether *Trim71* enrichment in the PE could restrain epicardial cell migration while promoting proliferation. To test this, we performed scratch wound-healing assays in Mec1 cells. Interestingly, *Trim71* inhibition triggered an early migratory response, with increased cell movement observed as early as 6 h after treatment and maintained also 12 h later, indicating that *Trim71* restrains epicardial cell migration (Figure 1D). Moreover, *Trim71* loss of function led to an increase in cell size without significant changes in Phalloidin intensity, suggesting morphological rearrangements that may facilitate the migratory process (Supplementary Figure S4A).

Together, these results identify *Trim71* as a PE-enriched gene that sustains epicardial cell proliferation while restraining cell migration. The downregulation of *Trim71* between E9.5 and E10.5, therefore, is consistent with the cellular switch from a highly proliferative state in the PE to a migratory phenotype required for epicardial expansion and maturation.

### 3.2. Trim71 Loss of Function Promotes Epicardial Lineage Gene Expression While Attenuating EMT and Vascular Programs

One of the main characteristics of the EE is its ability to invade the myocardium via EMT, subsequently serving as a source of cellular diversity by differentiating into CFs, SMCs, and ECs while depositing the extracellular matrix (ECM) required for proper cardiac development [7,41]. Given the observed effects of *Trim71* on epicardial proliferation and migration, we next investigated whether *Trim71* also influences epicardial lineage specification through loss-of-function experiments in Mec1 cells.

**Figure 2:**
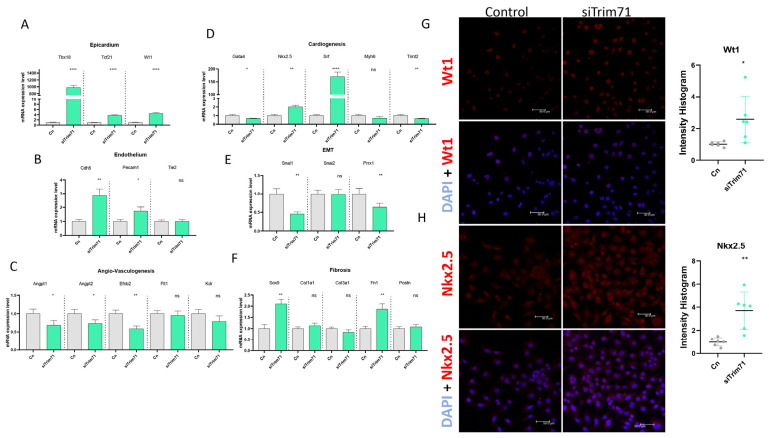
RT–qPCR analysis of cardiac markers. (**A**) Epicardial (*Tbx18*, *Tcf21*, *Wt1*), (**B**) endothelial (*Cdh5*, *Pecam1*, *Tie2*), and (**C**) angio-vasculogenesis (*Angpt1*, *Angpt2*, *Efnb2*, *Flt1*, *Kdr*) markers in Mec1 cells transfected with control siRNA (Cn) or siRNA targeting *Trim71* (si*Trim71*) (n = 3). *Trim71* inhibition resulted in increased expression of epicardial transcription factors, whereas angio-vasculogenic and endothelial markers showed gene-specific responses. RT–qPCR analysis of genes associated with (**D**) cardiogenic (*Gata4*, *Nkx2.5*, *Srf*, *Myh6*, *Tnnt2*), (**E**) EMT (*Snai1*, *Snai2*, *Prrx1*), and (**F**) matrix-associated/fibrosis-related genes (*Sox9*, *Col1a1*, *Col3a1*, *Fn1*) (n = 3). *Trim71* inhibition selectively altered the expression of a subset of cardiogenic EMT and matrix-associated genes, including increased expression of S*ox9* and *Fn1*, while collagen gene expression remained unchanged. (**G**,**H**) Representative images of the immunohistochemical detection of Wt1 and Nkx2.5 in control and si-Trim71 Mec1 cells, demonstrating increased expression at protein levels of these epicardial and cardiomyogenic markers, respectively. Scale bar represents 500 microns. Data are presented as mean ± SEM. Statistical significance is indicated (* *p* < 0.05, ** *p* < 0.01, **** *p* < 0.0001) (Cn: control).

*Trim71* inhibition resulted in increased expression of key epicardial markers, including transcription factors *Tbx18*, *Tcf21*, and *Wt1*, all of which are essential for proper epicardial formation and development (Figure 2A). We next examined the effect of *Trim71* loss of function on the expression of endothelial markers, including *Pecam1* and *Tie2*. Among these, *Pecam1* expression was specifically increased following *Trim71* inhibition (Figure 2B). The epicardium also plays a pivotal role in coronary vessel formation. In this context, *Trim71* inhibition negatively affected this process, resulting in reduced expression of the vascular-associated markers *Angpt1*, *Angpt22*, and *Efnb2*, while no significant effect was observed in *Flt1* and *Kdr* expression (Figure 2C).

We also evaluated the effect of *Trim71* loss of function on the expression of cardiogenic markers. *Trim71* inhibition resulted in increased expression of early cardiogenic markers such as *Nkx2.5* and *Srf*, key players in cardiomyocyte differentiation. However, a negative effect was observed on the expression of *Gata4* and *Tnnt2*, with no effect observed in *Myh6* (Figure 2D).

Furthermore, we also analyzed the expression of EMT markers such as *Snai1* and *Prrx1*, and both of them were downregulated upon *Trim71* inhibition; however, no significant effect was observed in *Snai2* expression. In contrast, *Cdh5*, an endothelial-enriched cadherin, displayed increased expression similarly to *Pecam* (Figure 2E–B). These results suggest that, in addition to regulating epicardial lineage identity, *Trim71* may be involved in maintaining epicardial cells in a state that restricts premature commitment toward an endothelial-like lineage. Finally, we assessed the expression of fibrosis-associated genes and observed that *Sox9* and *Fn1* were upregulated following *Trim71* inhibition (Figure 2F). *Fn1* encodes an ECM protein required for cell migration, whereas *Sox9* is a known regulator of fibrogenic processes. In contrast, no changes in the expression of *Col1a1*, *Col3a1*, and *Postn* were observed (Figure 2F). These data were validated at protein levels by immunofluorescent assays for *Wt1* (Figure 2G) and *Nkx2.5* (Figure 2H), observing an increased expression after the loss of function of Trim71.

Together, these results indicate that *Trim71* loss of function promotes the expression of epicardial determination genes while attenuating EMT and vascular-associated programs. This transcriptional profile supports a role for *Trim71* in maintaining epicardial cells in a progenitor-like state and limiting premature lineage commitment during epicardial development.

### 3.3. Reciprocal Regulation Between Trim71 and Let-7c/miR-30c During the PE to EE Transition

**Figure 3:**
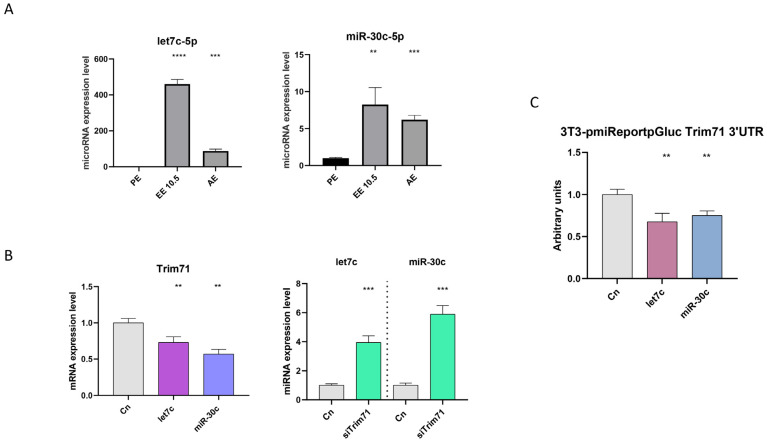
(**A**) RT–qPCR analysis of *let-7c-5p* and *miR-30c-5p* expression in proepicardium (PE), embryonic epicardium (EE), and adult epicardium (AE) (n = 3), showing increased expression in the EE compared with the PE and sustained expression in adulthood. (**B**) RT–qPCR analysis showing reduced *Trim71* expression following the gain of function of *let-7c* or *miR-30c* in Mec1 cells and increased *let-7c* and *miR-30c* expression after *Trim71* knockdown (n = 3), indicating a reciprocal negative feedback loop. (**C**) Luciferase reporter assays showing reduced activity of the *Trim71* 3′UTR upon overexpression of *let-7c* or *miR-30c* (n = 3), confirming direct post-transcriptional regulation. Data are presented as mean ± SEM. Statistical significance is indicated (** *p* < 0.01, and *** *p* < 0.001, **** *p* < 0.0001) (Cn: control).

Post-transcriptional regulation is fundamental for proper heart development, enabling the modulation of gene expression in response to developmental cues. Among post-transcriptional regulators, miRNAs play a central role by binding to the 3′UTRs of target mRNAs, thereby repressing their translation or promoting their degradation [42]. Our group has previously shown that the loss of function of *let-7c* and *miR-30c* leads to increased *Trim71* expression in both Mec1 cells and in primary epicardial cells isolated from E10.5 ventricles [30]. Both *let-7c* and *miR-30c* display differential upregulation in the EE at E10.5 compared to the PE, and they maintain detectable expression levels in the adult epicardium (Figure 3A). Additionally, *Trim71* expression is significantly reduced after the gain of function of either *let-7c* or *miR-30c*. Conversely, *Trim71* knockdown by siRNA resulted in the increased expression of both miRNAs, indicating the existence of a reciprocal negative feedback mechanism between *Trim71* and these miRNAs (Figure 3B).

To determine whether *let-7c* and *miR-30c* directly target *Trim71*, we analyzed the *Trim71* 3′UTR sequence and identified multiple conserved binding sites for the seed sequences of both miRNAs (Supplementary Figure S2C). This interaction was subsequently validated using a luciferase reporter assay in 3T3 cells, given that these cell types have basal *let-7c* or *miR-30c* expression levels. The overexpression of *let-7c* or *miR-30c* resulted in a significant reduction in luciferase activity, confirming that both miRNAs directly target the *Trim71* 3′UTR (Figure 3C).

Together, these results demonstrate that *Trim71* is directly regulated by *let-7c* and *miR-30c* through a post-transcriptional feedback loop. This regulatory axis provides a robust mechanism to fine-tune *Trim71* expression during epicardial development, supporting a dynamic balance between progenitor maintenance and differentiation-associated processes.

### 3.4. Let-7c and miR-30c Gain-of-Function Mimic Key Aspects of Trim71 Loss of Function in Epicardial Cells

**Figure 4:**
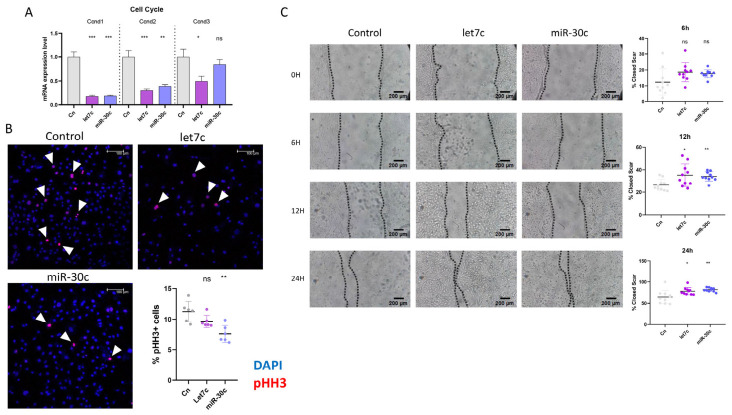
(**A**) RT–qPCR analysis of cell cycle gene markers (*Ccnd1*, *Ccnd2*, *Ccnd3*) in Mec1 cells transfected with control, *let-7c*, or *miR-30c* premiRs (n = 3), showing reduced expression of proliferation-associated markers. (**B**) Quantification of percentage of phospho-histone H3 (pHH3)-positive cells in Mec1 cells following *let-7c* or *miR-30c* overexpression, revealing decreased proliferative activity after *miR30-c* gain of function. Scale bars represent 100 microns. (**C**) Scratch wound-healing assays showing the percentage of wound closure at 6, 12, and 24 h after transfection with *let-7c* or *miR-30c* premiRs compared with control conditions (n = 10 fields per condition), indicating enhanced migratory capacity. Scale bars represent 200 microns. Data are presented as mean ± SEM. Statistical significance is indicated (* *p* < 0.05, ** *p* < 0.01, *** *p* < 0.001) (Cn: control).

Based on our previously obtained results, we next sought to evaluate whether these miRNAs could recapitulate the phenotypes observed upon *Trim71* loss of function. Notably, the gain of function of both *let-7c* and *miR-30c* led to a marked reduction in the expression of the cell cycle markers *Ccnd1* and *Ccnd2*, whereas *Ccnd3* expression was specifically decreased upon *let-7c* overexpression (Figure 4A). In addition, overexpression of *miR-30c* resulted in a reduced number of proliferative cells; conversely, the gain of function of *let 7c* did not produce statistically significant effects (Figure 4B), further supporting a decrease in proliferative activity. These effects are fully consistent with the impaired proliferative capacity observed following *Trim71* inhibition.

In line with our previous observations demonstrating a role for *let-7c* in the regulation of epicardial cell migration ex vivo [31], the overexpression of *let-7c*, as well as *miR-30c*, significantly promoted epicardial cell migration in vitro at 12 and 24 h (Figure 4C). We further analyzed whether the effect of *let-7c* and *miR-30c* gain of function could recapitulate the increased cell size observed after Trim71 inhibition in Mec1 cells. While no significant changes in cell area were found, a significant reduction in phalloidin staining intensity was detected after *let-7c* overexpression, indicating a decrease in actin polymerization or a reorganization of the actin cytoskeleton (Supplementary Figure S4B). This migratory response in Mec1 cells closely resembles the phenotype observed upon *Trim71* loss of function, further supporting a functional connection between these miRNAs and *Trim71* in the regulation of epicardial cell behavior.

**Figure 5:**
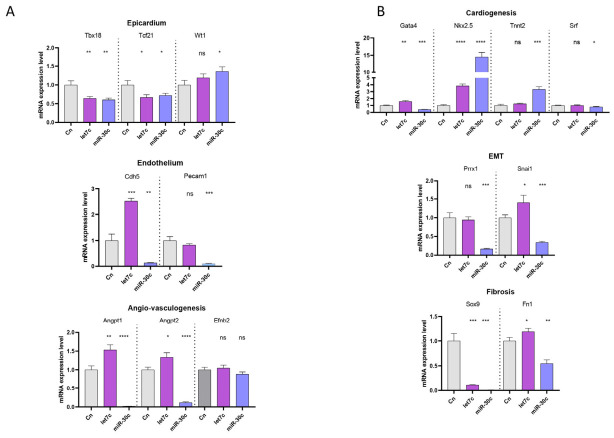
(**A**) RT–qPCR analysis of epicardial (*Tbx18*, *Tcf21*, *Wt1*), endothelial (*Pecam1*), angiogenic (*Angpt1*, *Angpt2*, *Efnb2*), and EMT (*Prrx1*, *Snai1*, *Cdh5*) markers in Mec1 cells transfected with control, *let-7c* or *miR-30c* premiRs (n = 3). miRNA overexpression resulted in selective and gene-specific modulation rather than uniform regulation across lineages. (**B**) RT–qPCR analysis of cardiogenic (*Gata4*, *Nkx2.5*, *Tnnt2*, *Srf*), EMT (*Prrx1*, *Snai1*), and fibrosis (*Sox9*, *Fn1*) markers (n = 3). *let-7c* and *miR-30c* displayed distinct and, in some cases, opposing regulatory effects, indicating selective modulation of mesenchymal and extracellular matrix-related components rather than induction of a generalized fibrotic or EMT program. Data are presented as mean ± SEM. Statistical significance is indicated (* *p* < 0.05, ** *p* < 0.01, *** *p* < 0.001, **** *p* < 0.0001) (Cn: control).

We next assessed whether the gain of function of *let-7c* and *miR-30c* was sufficient to recapitulate the effects of *Trim71* inhibition on epicardial lineage markers. While *Wt1* expression remained unchanged after *let-7c* modulation, it was significantly increased following *miR-30c* overexpression. In contrast, the expression of *Tbx18* and *Tcf21* was reduced after the gain of function of both miRNAs (Figure 5A). Regarding endothelial markers, *Cdh5* mRNA expression was increased after *let-7c* gain of function, in contrast with the negative effect observed in *miR-30c treatment.* Moreover, *let-7c* overexpression had no effect on *Pecam1* expression, whereas *miR-30c* gain of function resulted in a reduction of *Pecam1* levels (Figure 5A). A similar negative effect on the angio-vasculogenesis markers *Anggpt1* and *Anggpt2* was observed after *miR-30c* overexpression, resembling the response seen after *Trim71* inhibition. In contrast, these markers were increased following *let-7c* overexpression. Moreover, overexpression of both microRNAs has no effect on the expression of *Efnb2* (Figure 5A).

We subsequently analyzed the impact of miRNA overexpression on cardiogenic-related genes, including *Gata4*, *Nkx2.5*, *Tnnt2*, and *Srf*. A differential regulation of *Gata4* expression was observed, with upregulation after *let-7c* gain of function and downregulation following *miR-30c* overexpression, similarly to the effect observed after *Trim71* inhibition*. Nkx2.5* expression was increased upon gain of function of both miRNAs, consistent with *Trim71* loss of function, whereas *Tnnt2* expression was unaffected by *let-7c* but upregulated following *miR-30c* overexpression. In addition, *Srf* displays no significant modulation after *let-7c* gain of function, whereas overexpression of *miR-30c* decreases *Srf* expression (Figure 5B).

Concerning EMT-associated markers, *let-7c* overexpression positively regulated the expression of *Snai1* and *Cdh5*, while *Prrx1* levels remained unchanged. Conversely, *miR-30c* appeared to negatively regulate the EMT process, as indicated by reduced expression of *Snai1* and *Prrx1*, mirroring the effects observed upon *Trim71* inhibition (Figure 5B). Finally, fibrosis-associated markers, *Sox9* and *Fn1*, that were upregulated following *Trim71* inhibition displayed a differential modulation in response to miRNA overexpression. While *Sox9* expression was reduced in both miRNA gain-of-function conditions, *Fn1* expression was increased after *let-7c* overexpression and decreased following *miR-30c* treatment (Figure 5B).

Taken together, these results indicate that *let-7c* and *miR-30c* modulate key aspects of epicardial cell behavior that overlap with, but do not fully recapitulate, the effects observed after *Trim71* loss of function. Both miRNAs regulate epicardial proliferation and migration in a manner consistent with *Trim71* repression, supporting their functional interaction within a shared regulatory axis. However, their differential and sometimes opposing effects on epicardial, cardiogenic, endothelial, and EMT-associated markers suggest that miRNA-mediated control of *Trim71* represents only one layer of a broader regulatory network. These findings highlight the complexity of post-transcriptional regulation during epicardial maturation and point out *Trim71* as an integrative node for which its activity is fine-tuned by distinct miRNAs to coordinate multiple developmental programs. In summary, our results identify *Trim71* as a key regulator of epicardial cell behavior during the PE to EE transition. *Trim71* maintains epicardial cells in a proliferative, progenitor-like state while limiting migration and premature lineage commitment. This function is dynamically regulated by the miRNAs *let-7c* and *miR-30c* through a reciprocal feedback mechanism.

## 4. Discussion

Epicardial development is a highly dynamic and tightly regulated process that is essential for proper heart morphogenesis. Beyond serving as a progenitor source of cells, the embryonic epicardium functions as a signaling hub that orchestrates myocardial growth, coronary vessel formation, extracellular matrix deposition, and immune cell recruitment [8,15]. Consequently, precise temporal coordination of epicardial proliferation, migration, EMT, and lineage commitment is critical, and disruptions in these processes are known to result in congenital heart defects and adult cardiac pathologies [8,15,43].

In this study, we identify *Trim71* as a key regulator of the transition between the PE and the EE. *Trim71* is highly expressed in the PE, where epicardial progenitors expand, and it is downregulated as cells acquire migratory competence and mature into the EE. Functionally, inhibition of *Trim71* decreases epicardial cell proliferation while promoting migration, consistent with a role in maintaining a progenitor-like state. This behavior mirrors the *Trim71* functions described in other developmental contexts, particularly during early neurogenesis, where *Trim71* sustains progenitor proliferation and prevents premature differentiation [31,44,45]. Consistent with these observations, the decline in Trim71 expression during epicardial development coincides with the acquisition of the migratory capacity required for epicardial expansion [7].

Beyond its effects on proliferation and migration, *Trim71* also influences epicardial lineage-associated gene markers. Loss of *Trim71* resulted in increased expression of core epicardial transcription factors, including *Tbx18*, *Tcf21*, and *Wt1* while attenuating EMT- and vascular-associated gene expression. These findings suggest that *Trim71* contributes to maintaining epicardial cells in a progenitor-like state, preventing premature lineage commitment. Notably, although some cardiogenic and endothelial markers were modulated upon *Trim71* loss of function, the overall transcriptional profile indicates selective and context-dependent regulation. This reinforces the idea that *Trim71* does not impose a specific fate but instead modulates the preparation of epicardial cells to respond to developmental signals.

A central mechanism underlying these effects is the reciprocal regulation between *Trim71* and specific microRNAs. Members of the *let-7* family are well-established developmental regulators that promote differentiation and cell cycle progression in different contexts [46,47,48]. *Trim71* was originally identified as a conserved *let-7* target, forming a regulatory loop that balances progenitor maintenance and differentiation [49]. Moreover, *Trim71* has been shown to actively regulate miRNA pathways by promoting pre-let-7 degradation through interactions with LIN28 and TUT4, repressing mature *let-7* activity via RNA-dependent interactions with RISC components, and controlling the biogenesis of other miRNAs such as *miR-29a* [32,50]. These findings position *Trim71* as a broad modulator of post-transcriptional gene regulation rather than a simple miRNA target. In the epicardial context, we show that *let-7c* and *miR-30c* are upregulated in the EE compared to the PE, coinciding with *Trim71* downregulation. The gain of function of these miRNAs reduced *Trim71* expression and recapitulated key aspects of the *Trim71* loss-of-function phenotype, including reduced proliferation and enhanced migration. In addition, *Trim71* loss-of-function led to increased expression of both miRNAs, revealing a negative feedback loop. This reciprocal regulation provides a robust post-transcriptional mechanism to lower *Trim71* levels during the PE to EE transition, ensuring a gradual and coordinated shift from a proliferative to a migratory epicardial state. Notably, miRNA overexpression only partially mimicked *Trim71* loss of function regarding epicardial identity markers. While proliferation and migration were strongly affected, modulation of EMT, endothelial-associated, and extracellular matrix-related genes was more limited and, in some cases, divergent between *let-7c* and *miR-30c*. Current evidence shows that in other biological contexts, *let-7c* controls cell proliferation and migration by targeting *HoxB7*, *Hoxa1*, and *E2f5* [48,49,51]. Similarly, *miR-30c* has been reported as a regulator of *Sox9*, *Bcl2*, and *Mapk1* expression, thereby controlling cell migration and proliferation in different oncogenic settings [52,53,54]. This partial mimicry suggests that miRNA-mediated repression of *Trim71* controls epicardial maturation rather than acting as an on/off switch and that *Trim71* likely integrates additional regulatory inputs to coordinate multiple aspects of PE to EE progression. Such multilayered regulation may be particularly important during early epicardial development, where cells must maintain plasticity while responding to rapidly changing spatial and temporal cues.

It is important to acknowledge the limitations of this study. Our conclusions are primarily based on in vitro analyses using Mec1 epicardial cell lines. While this model enables precise manipulation of gene expression and dissection of cell-autonomous mechanisms governing PE- to EE-like transitions, it cannot fully recapitulate the complex tissue interactions and biomechanical forces present in vivo. Nevertheless, the consistency of our findings with established roles of *Trim71* and miRNA-mediated regulation in other developmental systems supports their physiological relevance. Future in vivo studies will be required to determine how the *Trim71*–miRNA axis integrates with myocardial-derived signals to control epicardial development and cardiac morphogenesis.

**Figure 6:**
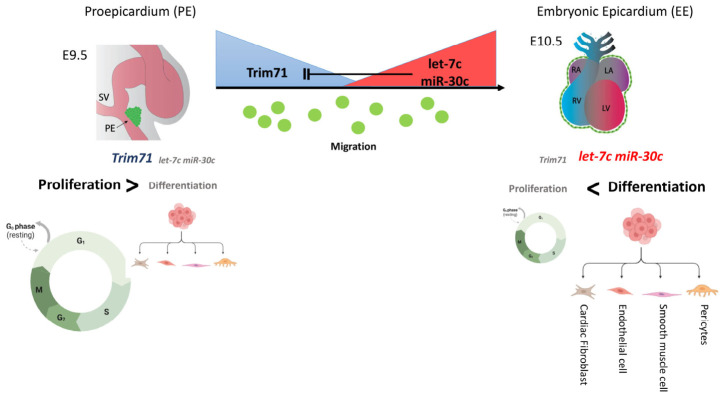
Schematic model summarizing the molecular mechanisms governing the PE-to-EE transition. In PE, high *Trim71* expression maintains proepicardial cells in a proliferative, progenitor-like state. As development progresses, during epicardial maturation, increased expression of *let-7c* and *miR-30c* represses *Trim71*, leading to reduced proliferation, enhanced migratory competence, and transcriptional priming characteristic of the formation and expansion of the embryonic epicardium (EE).

In conclusion, our results support a model in which *Trim71* functions as a temporal regulator of epicardial maturation during the PE (E9.5) to EE (E10.5) transition. High *Trim71* expression in the PE sustains progenitor expansion and limits premature migration by restraining epicardial cell commitment observed in the regulation of epicardial gene markers. The repression of *Trim71* by *let-7c* and *miR-30c* induces migratory competence, which is essential for proper epicardial development. This regulatory axis ensures that epicardial cells reach the myocardial surface at the appropriate developmental stage, preserving both progenitor pool size and functional flexibility (Figure 6). Nonetheless, it is important to highlight that the usage of Mec1 epicardial cells, a cell line derived from E13.5 mouse ventricular epicardial explants, might limit the extrapolation of these results in vivo, since it likely models EE cells. Therefore, additional experiments will be required in vivo to validate this regulatory axis.

## Data Availability

The original contributions presented in this study are included in this article/Supplementary Materials. Further inquiries can be directed to the corresponding author.

## Supplementary Materials

The following supporting information can be downloaded at https://www.mdpi.com/article/10.3390/jcdd13060237/s1.

## References

[1] Quijada P., Trembley M.A., Small E.M. (2020). The Role of the Epicardium during Heart Development and Repair. Circ. Res..

[2] Masters M., Riley P.R. (2014). The epicardium signals the way towards heart regeneration. Stem Cell Res..

[3] Limana F., Capogrossi M.C., Germani A. (2011). The epicardium in cardiac repair: From the stem cell view. Pharmacol. Ther..

[4] Serluca F.C. (2008). Development of the proepicardial organ in the zebrafish. Dev. Biol..

[5] Jahr M., Schlueter J., Brand T., Männer J. (2008). Development of the proepicardium in *Xenopus laevis*. Dev. Dyn..

[6] Fransen M.E., Lemanski L.F. (1990). Epicardial development in the axolotl, *Ambystoma mexicanum*. Anat. Rec..

[7] Cao Y., Duca S., Cao J. (2020). Epicardium in Heart Development. Cold Spring Harb. Perspect. Biol..

[8] Carmona R., Guadix J.A., Cano E., Ruiz-Villalba A., Portillo-Sánchez V., Pérez-Pomares J.M., Muñoz-Chápuli R. (2010). The embryonic epicardium: An essential element of cardiac development. J. Cell Mol. Med..

[9] Lamouille S., Xu J., Derynck R. (2014). Molecular mechanisms of epithelial–mesenchymal transition. Nat. Rev. Mol. Cell Biol..

[10] Pérez-Pomares J.M., Carmona R., González-Iriarte M., Atencia G., Wessels A., Muñoz-Chápuli R. (2002). Origin of coronary endothelial cells from epicardial mesothelium in avian embryos. Int. J. Dev. Biol..

[11] Mikawa T., Gourdie R.G. (1996). Pericardial Mesoderm Generates a Population of Coronary Smooth Muscle Cells Migrating into the Heart along with Ingrowth of the Epicardial Organ. Dev. Biol..

[12] Cao J., Poss K.D. (2018). The epicardium as a hub for heart regeneration. Nat. Rev. Cardiol..

[13] Simões F.C., Riley P.R. (2018). The ontogeny, activation and function of the epicardium during heart development and regeneration. Development.

[14] Tian X., Pu W.T., Zhou B. (2015). Cellular Origin and Developmental Program of Coronary Angiogenesis. Circ. Res..

[15] Lie-Venema H., van den Akker N.M.S., Bax N.A.M., Winter E.M., Maas S., Kekarainen T., Hoeben R.C., deRuiter M.C., Poelmann R.E., Gittenberger-de Groot A.C. (2007). Origin, Fate, and Function of Epicardium-Derived Cells (EPDCs) in Normal and Abnormal Cardiac Development. Sci. World J..

[16] Gittenberger-de Groot A.C., Winter E.M., Goumans M.J., Bartelings M.M., Poelmann R.E. (2016). The Arterial Epicardium: A Developmental Approach to Cardiac Disease and Repair. Etiology and Morphogenesis of Congenital Heart Disease.

[17] Briggs L.E., Kakarla J., Wessels A. (2012). The pathogenesis of atrial and atrioventricular septal defects with special emphasis on the role of the dorsal mesenchymal protrusion. Differentiation.

[18] Gittenberger-de Groot A.C., Vrancken Peeters M.P.F.M., Bergwerff M., Mentink M.M.T., Poelmann R.E. (2000). Epicardial Outgrowth Inhibition Leads to Compensatory Mesothelial Outflow Tract Collar and Abnormal Cardiac Septation and Coronary Formation. Circ. Res..

[19] Kathiriya I.S., Nora E.P., Bruneau B.G. (2015). Investigating the transcriptional control of cardiovascular development. Circ. Res..

[20] Greulich F., Farin H.F., Schuster-Gossler K., Kispert A. (2012). Tbx18 function in epicardial development. Cardiovasc. Res..

[21] von Gise A., Zhou B., Honor L.B., Ma Q., Petryk A., Pu W.T. (2011). WT1 regulates epicardial epithelial to mesenchymal transition through β-catenin and retinoic acid signaling pathways. Dev. Biol..

[22] Grieskamp T., Rudat C., Lüdtke T.H.W., Norden J., Kispert A. (2011). Notch Signaling Regulates Smooth Muscle Differentiation of Epicardium-Derived Cells. Circ. Res..

[23] Leung O., Zhou B., Lui K. (2016). Vascular Development and Regeneration in the Mammalian Heart. Dev. Dis..

[24] Sayed D., Abdellatif M. (2011). MicroRNAs in Development and Disease. Physiol. Rev..

[25] Jonas S., Izaurralde E. (2015). Towards a molecular understanding of microRNA-mediated gene silencing. Nat. Rev. Genet..

[26] Treiber T., Treiber N., Meister G. (2012). Regulation of microRNA biogenesis and function. Thromb. Haemost..

[27] Krol J., Loedige I., Filipowicz W. (2010). The widespread regulation of microRNA biogenesis, function and decay. Nat. Rev. Genet..

[28] Zilberman D., Cao X., Jacobsen S.E. (2003). *ARGONAUTE4* Control of Locus-Specific siRNA Accumulation and DNA and Histone Methylation. Science.

[29] Singh M.K., Lu M.M., Massera D., Epstein J.A. (2011). MicroRNA-processing Enzyme Dicer Is Required in Epicardium for Coronary Vasculature Development. J. Biol. Chem..

[30] Castillo-Casas J.M., Dueñas Á., Hernández-Torres F., Carmona R., Muñoz-Chápuli R., Dopazo A., Álvarez R., de Luis E.V., Aranega A.E., Franco D. (2025). Foxf1-mediated co-regulation of miR-495 and let-7c modulates epicardial cell migration and myocardial specification. Cell. Mol. Life Sci..

[31] Loedige I., Gaidatzis D., Sack R., Meister G., Filipowicz W. (2013). The mammalian TRIM-NHL protein TRIM71/LIN-41 is a repressor of mRNA function. Nucleic Acids Res..

[32] Treiber T., Treiber N., Plessmann U., Harlander S., Daiß J.L., Eichner N., Lehmann G., Schall K., Urlaub H., Meister G. (2017). A Compendium of RNA-Binding Proteins that Regulate MicroRNA Biogenesis. Mol. Cell.

[33] Lozano-Velasco E., Vallejo D., Esteban F.J., Doherty C., Hernández-Torres F., Franco D., Aránega A.E. (2015). A *Pitx2*-MicroRNA Pathway Modulates Cell Proliferation in Myoblasts and Skeletal-Muscle Satellite Cells and Promotes Their Commitment to a Myogenic Cell Fate. Mol. Cell Biol..

[34] Bustin S.A., Benes V., Garson J.A., Hellemans J., Huggett J., Kubista M., Mueller R., Nolan T., Pfaffl M.W., Shipley G.L. (2009). The MIQE Guidelines: Minimum Information for Publication of Quantitative Real-Time PCR Experiments. Clin. Chem..

[35] Livak K.J., Schmittgen T.D. (2001). Analysis of Relative Gene Expression Data Using Real-Time Quantitative PCR and the 2−ΔΔCT Method. Methods.

[36] Ascione F., Vasaturo A., Caserta S., D’Esposito V., Formisano P., Guido S. (2016). Comparison between fibroblast wound healing and cell random migration assays in vitro. Exp. Cell Res..

[37] Toro R., Pérez-Serra A., Mangas A., Campuzano O., Sarquella-Brugada G., Quezada-Feijoo M., Ramos M., Alcalá M., Carrera E., García-Padilla C. (2022). miR-16-5p Suppression Protects Human Cardiomyocytes against Endoplasmic Reticulum and Oxidative Stress-Induced Injury. Int. J. Mol. Sci..

[38] García-Padilla C., Lozano-Velasco E., García-López V., Aránega A., Franco D., García-Martínez V., López-Sánchez C. (2024). miR-1 as a Key Epigenetic Regulator in Early Differentiation of Cardiac Sinoatrial Region. Int. J. Mol. Sci..

[39] Bonet F., Dueñas Á., López-Sánchez C., García-Martínez V., Aránega A.E., Franco D. (2015). MiR-23b and miR-199a impair epithelial-to-mesenchymal transition during atrioventricular endocardial cushion formation. Dev. Dyn..

[40] Torres-Fernández L.A., Mitschka S., Ulas T., Weise S., Dahm K., Becker M., Händler K., Beyer M., Windhausen J., Schultze J.L. (2021). The stem cell–specific protein TRIM71 inhibits maturation and activity of the prodifferentiation miRNA let-7 via two independent molecular mechanisms. RNA.

[41] Carmona R., Barrena S., López Gambero A.J., Rojas A., Muñoz-Chápuli R. (2020). Epicardial cell lineages and the origin of the coronary endothelium. FASEB J..

[42] Lozano-Velasco E., Garcia-Padilla C., del Mar Muñoz-Gallardo M., Martinez-Amaro F.J., Caño-Carrillo S., Castillo-Casas J.M., Sanchez-Fernandez C., Aranega A.E., Franco D. (2022). Post-Transcriptional Regulation of Molecular Determinants during Cardiogenesis. Int. J. Mol. Sci..

[43] Sanchez-Fernandez C., Rodriguez-Outeiriño L., Matias-Valiente L., Ramírez de Acuña F., Franco D., Aránega A.E. (2023). Understanding Epicardial Cell Heterogeneity during Cardiogenesis and Heart Regeneration. J. Cardiovasc. Dev. Dis..

[44] Duy P.Q., Jux B., Zhao S., Mekbib K.Y., Dennis E., Dong W., Nelson-Williams C., Mehta N.H., Shohfi J.P., Juusola J. (2024). *TRIM71* mutations cause a neurodevelopmental syndrome featuring ventriculomegaly and hydrocephalus. Brain.

[45] Duy P.Q., Furey C.G., Kahle K.T. (2019). Trim71/lin-41 Links an Ancient miRNA Pathway to Human Congenital Hydrocephalus. Trends Mol. Med..

[46] Liu H.Y., Huang C.M., Hung Y.F., Hsueh Y.P. (2015). The microRNAs Let7c and miR21 are recognized by neuronal Toll-like receptor 7 to restrict dendritic growth of neurons. Exp. Neurol..

[47] Cai L., Wang Z., Zheng H., Xu L. (2020). The let-7c/HoxB7 axis regulates the cell proliferation, migration and apoptosis in hepatocellular carcinoma. Anti-Cancer Drugs.

[48] Zhan M., Qu Q., Wang G., Liu Y.Z., Tan S.L., Lou X.Y., Yu J., Zhou H.H. (2013). Let-7c Inhibits NSCLC Cell Proliferation by Targeting HOXA1. Asian Pac. J. Cancer Prev..

[49] Liu Q., Chen X., Novak M.K., Zhang S., Hu W. (2021). Repressing Ago2 mRNA translation by Trim71 maintains pluripotency through inhibiting let-7 microRNAs. eLife.

[50] Yin J., Kim T.H., Park N., Shin D., Choi H.I., Cho S., Park J.B., Kim J.H. (2016). TRIM71 suppresses tumorigenesis via modulation of Lin28B-let-7-HMGA2 signaling. Oncotarget.

[51] Huang M., Gong X. (2018). Let-7c Inhibits the Proliferation, Invasion, and Migration of Glioma Cells via Targeting E2F5. Oncol. Res. Featur. Preclin. Clin. Cancer Ther..

[52] Yuan L.Q., Zhang T., Xu L., Han H., Liu S.H. (2021). miR-30c-5p inhibits glioma proliferation and invasion via targeting Bcl2. Transl. Cancer Res..

[53] Zhang A., Liu X., Wang J., Deng K., Wang J. (2021). MiR-30c-5p Directly Targets MAPK1 to Regulate the Proliferation, Migration and Invasion of Adenomyotic Epithelial Cells in Adenomyosis. Twin Res. Hum. Genet..

[54] Liu S., Li X., Zhuang S. (2019). miR-30c Impedes Glioblastoma Cell Proliferation and Migration by Targeting SOX9. Oncol. Res. Featur. Preclin. Clin. Cancer Ther..

