# Supplementary material for: RNA-Binding Protein Trim71 Controls Epicardial Cell Migration
Source: J Cardiovasc Dev Dis. 2026 May 31;13(6):237. doi: 10.3390/jcdd13060237 (PMC13302136; doi:10.3390/jcdd13060237)
Supplement: Supplementary file 1 [file jcdd-13-00237-s001.zip › jcdd-4186972-Table S1.pdf]

mRNA primer sequences	
Trim71_Fw	CTGATGGTGCTGTCCAGCTT
Trim71_Rv	GGTCCAGTCTGAGGTCAAGG
GATA4 Fw	TCTCACTATGGGCACAGCAG
GATA4 Rv	CGAGCAGGAATTTGAAGAGG
Myh6_Fw	AGGACCTGGTGGACAAGCTA
Myh6_Rv	CTGCACCTTGCGGAACTT
Nkx2.5 Fw	TTGGCGTCGGGGACTTGAAC
Nkx2.5 Rv	GGTGGGTGTGAAATCCGAGGGAC
Tnnt2 Fw	TTCGACCTGCAGGAAAAGTT
Tnnt2 Rv	GCACAGCTTTGACGAGAACA
Srf Fw	GCTCAATGCCTTCTCTCAGG
Srf Rv	CCCTATCACAGCCATCTGGT
TcF21 Fw	CGCTCACTTAAGGCAGATCC
Tct21 Rv	TCACCACTTCCTTCAGGTCA
Tbx18 Fw	GGCCATCATTACGGACTCTC
Tbx18 Rv	GGAGCAAGAGGATCCAGACA
Wt1 Fw	ACCATCTGAAGACCCACACC
Wt1 Rv	TCTGATGCATGTTGTGATGG
Pecam1 Fw	AGCCTAGTGTGGAAGCCAAC
Pecam1 Rv	CTCAAGGGAGGACACTTCCA
Postn Fw	AGCCAACAAAAGGGTTCAAG
Postn Rv	TGAAGTCAGTGTGGTGGCTCT
Tie2 Fw	AACAGGATGCTGGAAGAACG
Tie2 Rv	GAGGAGAAATGGCCGTTGTA
Angpt1 Fw	GATCTTACACGGTGCCGATT
Angpt1 Rv	TTAGATTGGAAGGGCCACAG
Angpt2 Fw	CAGCCAACCAGGAAGGAGGTGATT
Angpt2 Rv	AAGTTGGAAGGACCACATGC
Efnb2 Fw	AGGAATCACGGTCCAACAAG
Efnb2 Rv	GGAACCCAGGAGATTGTTCC
Flt 1 Fw	ATCCCAGCAGCAACTTAGGA
Flt 1 Rv	GAGAGTCAGCCACCACCAAT
Cdh5 Fw	TGCATCCTCACCATCACAGT
Cdh5 Rv	AGTGACCAACTGCTCGTGAA
Prrx1 Fw	GCAGGACAATGACAAGTTGA
Prrx1 Rv	GAACAAAAGCATCCGGGTAA
Snai1 Fw	CTTGTGTCTGCACGACCTGT
Snai 1 Rv	AGTGGGAGCAGGAGAATGG
snai2 Fw	CATTGCCTTGTGTCTGCAAG
Snai2 Rv	GATGTGCCCTCAGGTTTGAT
Col1a1 Fw	CACTGCAAGAACAGCGTAGC
Col1a1 Rv	GACTGTCTTGCCCCAAGTTC
Col3a1 Fw	AGGCCAGTGGCAATGTAAAG
Col3a1 Rv	ATTGCCTTGCGTGTTTGATA
Sox9 Fw	GACTCCCCACATTCCTCCTC
Sox9 Rv	CCCTCTCGCTTCAGATCAAC
Gapdh_Fw	GGCATTGCTCTCAATGACAA
Gapdh_Rv	TGTGAGGGAGATGCTCAGTG

	3'UTR primer sequences
3'UTR Trim71 fw	GGACTAGTTTGCCTAAGTCCTACCTCAGC
3'UTR Trim71 Rv	GGGAAGCTTAGCAAGCCACAGCCATATCT

siRNA sequences	
siTrim71 5'	GACAAAGACAAUCAUCGCAUU
siTrim71 3'	UUCUGUUUCUGUUAGUAGCGU
Scramble 5'	AUAACAAUUAAAGUCUGAUACACCACA
Scramble 3'	UGUGGUGUAUCAGACUUUAAUUGUUAU
