# Supplementary figures and images for: RNA-Binding Protein Trim71 Controls Epicardial Cell Migration
Source: J Cardiovasc Dev Dis. 2026 May 31;13(6):237. doi: 10.3390/jcdd13060237 (PMC13302136; doi:10.3390/jcdd13060237)

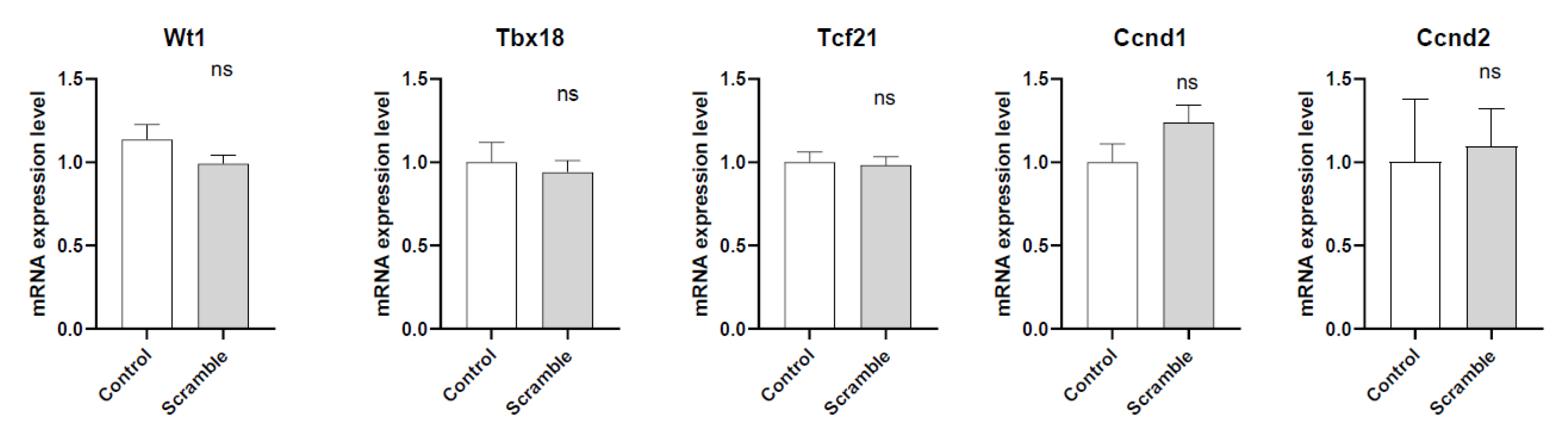

Supplement: Supplementary file 1 [file jcdd-13-00237-s001.zip › Supplementary Figure S1.tif]

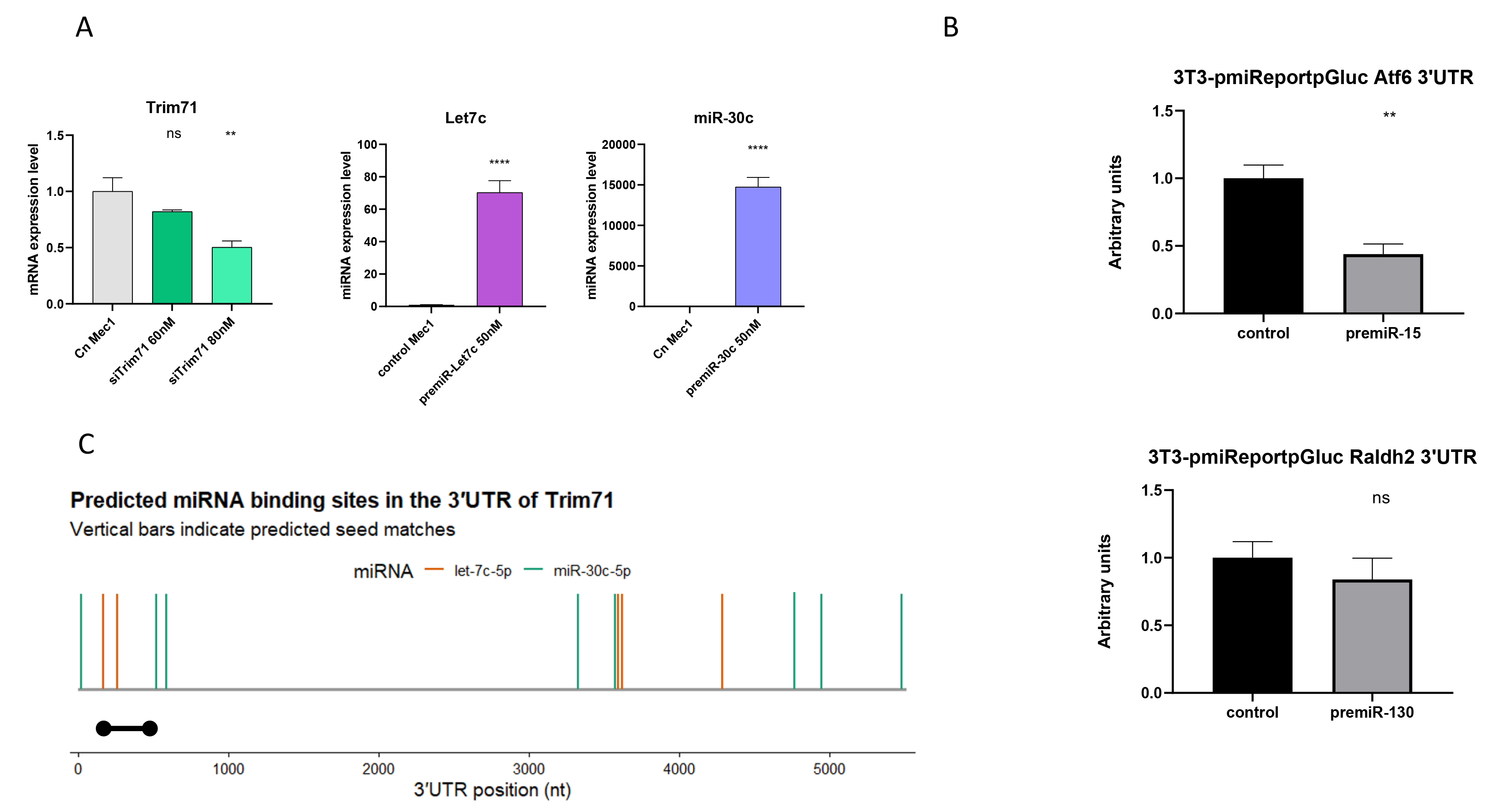

Supplement: Supplementary file 1 [file jcdd-13-00237-s001.zip › Supplementary Figure S2.tif]

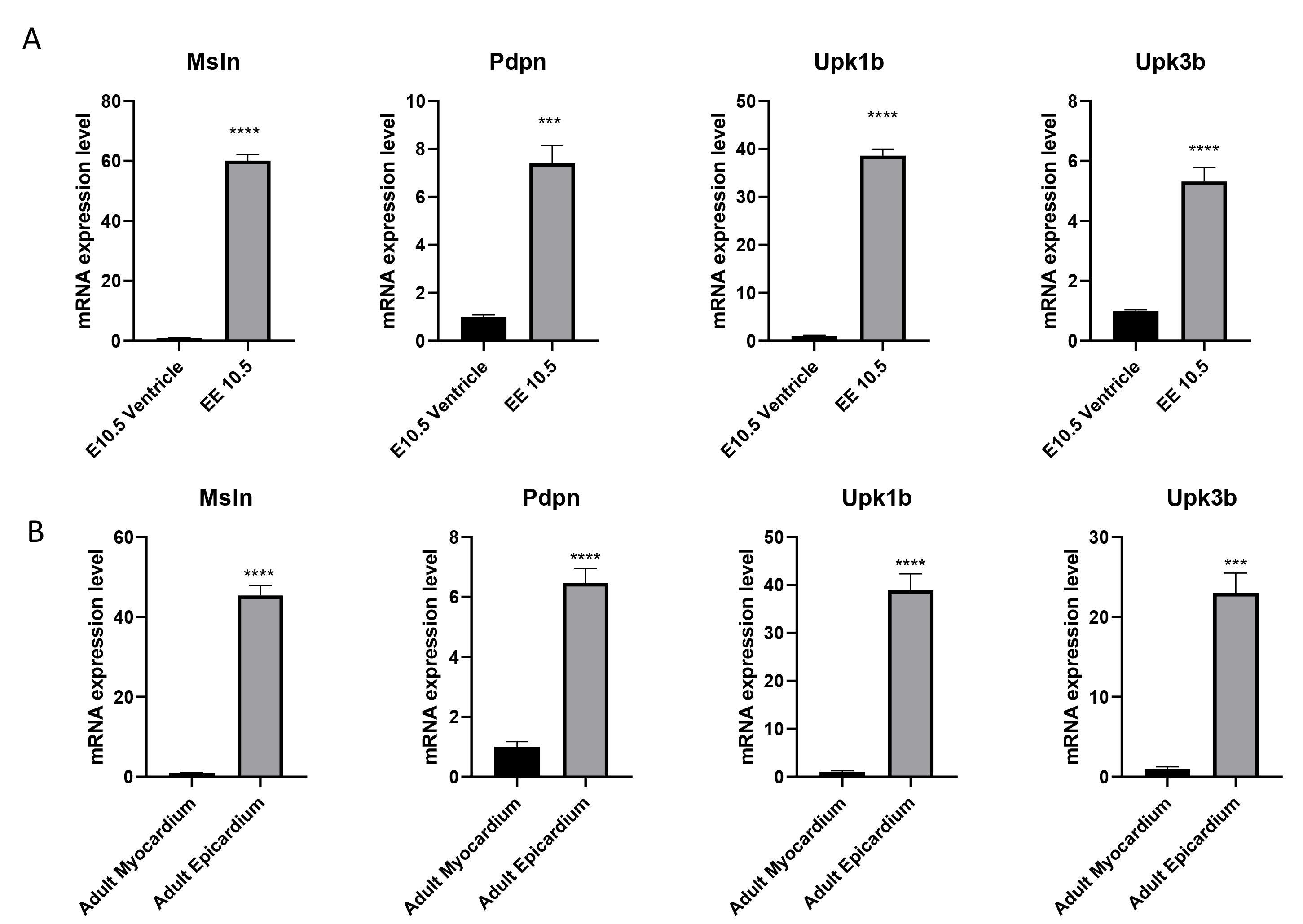

Supplement: Supplementary file 1 [file jcdd-13-00237-s001.zip › Supplementary Figure S3.tif]

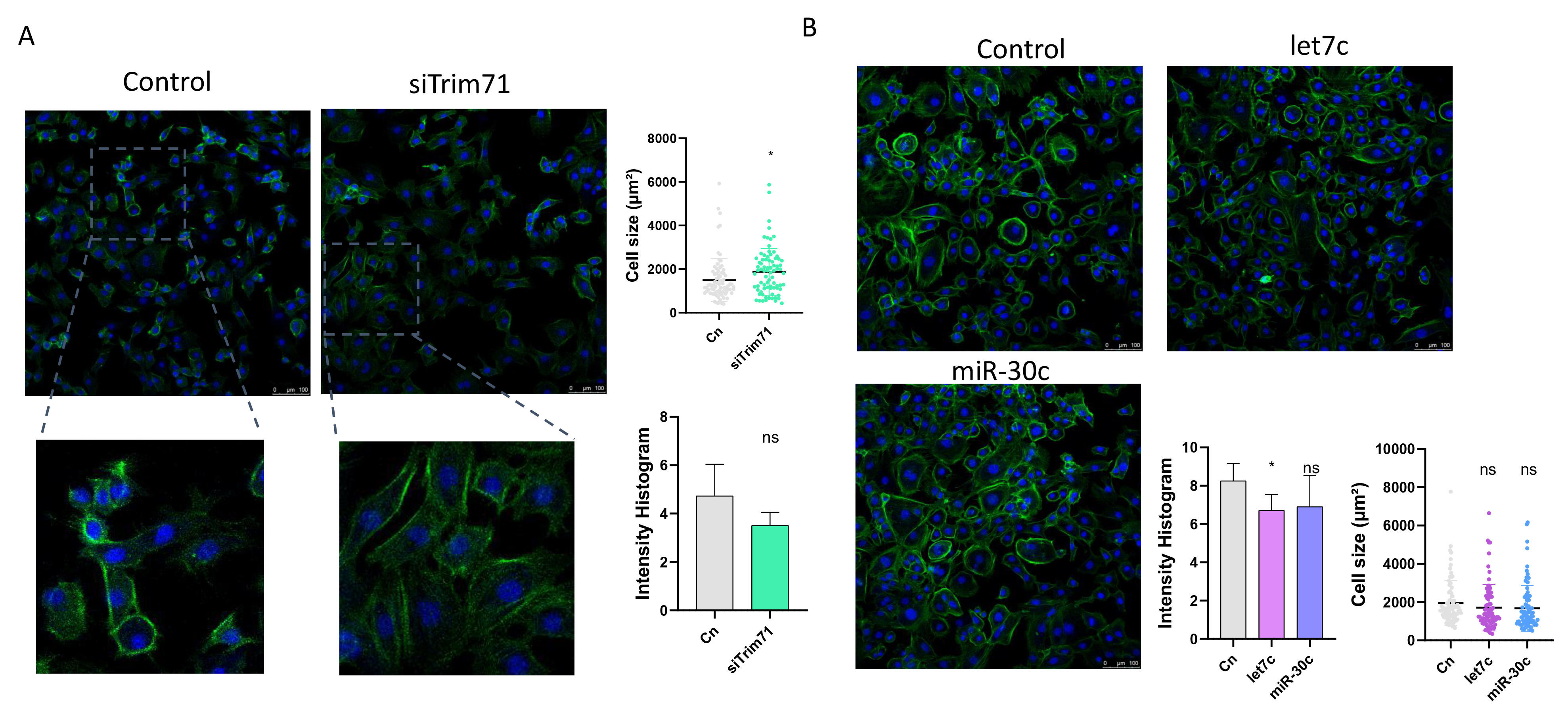

Supplement: Supplementary file 1 [file jcdd-13-00237-s001.zip › Supplementary Figure S4.tif]
